# A common cause of sudden and thunderclap headaches: reversible cerebral vasoconstriction syndrome

**DOI:** 10.1186/1129-2377-15-13

**Published:** 2014-03-01

**Authors:** Yu-Chen Cheng, Kuei-Hong Kuo, Tzu-Hsien Lai

**Affiliations:** 1 Section of Neurology, Department of Internal Medicine, Far Eastern Memorial Hospital, No. 21, Sec. 2, Nanya S. Rd., Ban-Chiao Dist., New Taipei City 220, Taiwan; 2 Department of Radiology, Far Eastern Memorial Hospital, New Taipei, Taiwan; 3 Department of Neurology, Neurological Institute, Taipei Veterans General Hospital, Taipei, Taiwan; 4 Department of Neurology, National Yang-Ming University School of Medicine, Taipei, Taiwan

**Keywords:** Bath-related thunderclap headache, Orgasmic headache, Primary cough headache, Primary exertional headache, Primary headache associated with sexual activity, Reversible cerebral vasoconstriction syndrome, Sentinel headache, Sudden headache, Subarachnoid hemorrhage, Thunderclap headache

## Abstract

**Background:**

Thunderclap headache (TCH) is a sudden headache (SH) with accepted criteria of severe intensity and onset to peak within one minute. It is a well-known presentation for subarachnoid hemorrhage (SAH) but most patients with TCH or SH run a benign course without identifiable causes. Reversible cerebral vasoconstriction syndrome (RCVS), a recently recognized syndrome characterized by recurrent TCH attacks, has been proposed to account for most of these patients.

**Methods:**

We recruited consecutive patients presenting with SH at our headache clinic. Computed tomography and/or magnetic resonance imaging with angiography were performed to exclude structural causes and to identify vasoconstriction. Catheter angiography and lumbar puncture were performed with patients consent. Reversibility of vasoconstriction was confirmed by follow-up study.

**Results:**

From July 2010 to June 2013, 31 patients with SH were recruited. Twenty-four (72.7%) of these SH patients exhibited headache fulfilling the TCH criteria. The diagnosis of RCVS was confirmed in 14 (45.2%) of patients with SH and 11 (45.8%) of patients with TCH. Other diagnoses were as follows: primary headaches (SH: 41.9%, TCH: 45.8%) and other secondary causes (SH: 12.9%, TCH: 8.3%). Compared with non-RCVS patients, patients with RCVS were older (50.8 ± 9.3 years vs. 40.8 ± 10.0 years, *P* = 0.006) and less likely to experience short headache duration of < 1 hour (23.1% vs. 78.6%, *P* = 0.007). Patients with RCVS were more likely to cite bathing (42.9% vs. 0%, *P* = 0.004) and less likely to cite exertion (0% vs. 29.4%, *P* = 0.048) as headache triggers.

**Conclusions:**

Reversible cerebral vasoconstriction syndrome is a common cause of SH and TCH. Considering the potential mortality and morbidity of RCVS, systemic examination of cerebral vessels should be performed in these patients.

## Background

Thunderclap headache (TCH) is a well-known presentation of subarachnoid hemorrhage (SAH). The term was first used in a patient who had three episodes of “intense sentinel headache of sudden onset” before an unruptured aneurysm was found [1]. A later study following 71 patients for an average of 3.3 years reported no SAH, which led to the concept of “benign TCH” [2]. Other studies recruiting patients with similar sudden headache (SH) showed that 6-25% of these patients exhibited SAH [3-5]. More than one-half of the patients in these studies did not receive a definite diagnosis. The term “TCH” has been defined as a SH fulfilling criteria of both severe intensity and onset to peak within one minute [6]. Several possible causes of TCH have been reported, ranging from various vascular lesions of the brain to indolent headaches associated with sexual activity (HSA) or other triggers [7]. Nonetheless, most patients with SH are still categorized as having a “benign headache” [5].

Recently, reversible cerebral vasoconstriction syndrome (RCVS) has been added to the list of TCH differential diagnoses [8]. RCVS is a unifying term which encompasses a group of recurrent headache syndromes including: Call-Fleming syndrome, thunderclap headache with reversible vasospasm, benign angiopathy of the central nervous system, postpartum cerebral angiopathy, among others [8]. The clinical presentation of RCVS is characterized by recurrent TCH attacks and multifocal vasoconstriction which resolves within 2 to 3 months [8,9]. In contrast to “benign” causes of TCH, RCVS is linked with clinical (focal neurological deficits and seizure) and radiological (cortical SAH, intracranial hemorrhage, ischemic stroke, arterial dissection and posterior reversible encephalopathy syndrome) abnormalities, and sometimes increased morbidity and mortality [9-11]. After the term “RCVS” was coined in 2007, reports of this condition began accumulating rapidly, though most of these reports are sporadic cases. It has been postulated that RCVS is under-recognized and “accounts for most benign TCH” [12,13]. We aimed to test the hypothesis of RCVS as a common cause in patients presenting with TCH and similar SH.

## Methods

### Clinical settings

We recruited consecutive patients with SH at our headache clinic in Far Eastern Memorial Hospital from July 2010 to June 2013. The headache clinic has been operating since August 2009. Far Eastern Memorial Hospital is a 1012-bed medical center in Taipei, Taiwan. Under the local health care system, patients are free to call upon any hospital without a referral. Our patients are either self-referred or referred by our colleague neurologists. All patients presenting at this headache clinic are requested to fill out a detailed headache intake form, have their medical and headache histories recorded, receive a neurological examination and are suggested to keep a headache diary.

### Diagnostic algorithm

Following previous studies, patients presenting with SH suggesting possible vascular origin, especially sentinel leakage or SAH, were included [3,4]. Patients were eligible for the study if they presented with a new type of headache within one month of onset and the duration of headache was at least > 1 minute. Patients with symptoms or signs indicating other primary (e.g. cranial autonomic symptoms, headache always occurring during sleep) or secondary causes of headache (e.g. trauma, meningitis, sinusitis) were excluded. Thunderclap headache was defined following the ICHD-2 (International Classification of Headache Disorders, 2nd edition) criteria of onset to peak within one minute and severe intensity (≥7 on a 0–10 numerical rating scale) [6]. Patients were recorded as having TCH if they reported at least one, but not every, attack fulfilling the criteria. Neuroimaging studies, including brain computed tomography (CT) and/or magnetic resonance imaging (MRI) with 3-dimensional reformatting angiography (CTA and/or MRA), were arranged to exclude structural causes and to identify vasoconstriction. Catheter angiography and lumbar puncture were performed if patients agreed. All imaging data were interpreted by a neuroradiologist (Kuo KH), who was blinded to the diagnoses. The diagnosis of RCVS was made following ICHD-2 for headache attributed to benign (or reversible) angiopathy of the central nervous system (code 6.7.3) (Table 1) [6]. Follow-up neuroimaging studies (CTA or MRA) were performed approximately 3 months later to confirm the reversibility of vasoconstriction. Diagnoses of other primary headache disorders were made following ICHD-2, including primary cough headache (code 4.2), primary exertional headache (code 4.3), primary headache associated with sexual activity (HSA) (code 4.4), and primary TCH (code 4.7) [6].

**Table 1:** Diagnostic criteria of RCVS

	
ICHD-2 (code 6.7.3) [6]	Headache attributed to benign (or reversible) angiopathy of the CNS
	A. Diffuse, severe headache of abrupt or progressive onset, with or without focal neurological deficits and/or seizures and fulfilling criteria C and D
	B. ‘Strings and beads’ appearance on angiography and SAH ruled out by appropriate investigations
	C. One or both of the following:
	1. headache develops simultaneously with neurological deficits and/or seizures
	2. headache leads to angiography and discovery of ‘strings and beads’ appearance
	D. Headache (and neurological deficits, if present) resolves spontaneously within 2 months
ICHD-3, beta version (code 6.7.3) [14]	Headache attributed to RCVS
	A. Any new headache fulfilling criterion C
	B. RCVS has been diagnosed
	C. Evidence of causation demonstrated by at least one of the following:
	1. headache, with or without focal deficits and/or seizures, has led to angiography (with ‘strings and beads’ appearance) and diagnosis of RCVS 2. headache has either or both of the following characteristics:
	a) recurrent during ≤1 month, and with thunderclap onset
	b) triggered by sexual activity, exertion, Valsalva maneuvers, emotion, bathing and/or showering
	3. no new significant headache occurs >1 month after onset
	D. Not better accounted for by another ICHD-3 diagnosis, and aneurysmal SAH has been excluded by appropriate investigations.
ICHD-3, beta version (code 6.7.3.1) [14]	Headache probably attributed to RCVS
	A. Any new headache fulfilling criterion C
	B. RCVS is suspected, but cerebral angiography is normal
	C. Probability of causation demonstrated by all of the following:
	1. at least two headaches within 1 month, with all three of the following characteristics:
	a) thunderclap onset, and peaking in <1 minute
	b) severe intensity
	c) lasting ≥5 minutes
	2. at least one thunderclap headache has been triggered by one of the following:
	a) sexual activity (just before or at orgasm)
	b) exertion
	c) Valsalva-like maneuver
	d) emotion
	e) bathing and/or showering
	f) bending
	3. no new thunderclap or other significant headache occurs >1 month after onset
	D. Not fulfilling ICHD-3 criteria for any other headache disorder
	E. Not better accounted for by another ICHD-3 diagnosis, and aneurysmal SAH has been excluded by appropriate investigations.

ICHD: International Classification of Headache Disorders; CNS: central nervous system; RCVS: reversible cerebral vasoconstriction syndrome; SAH: subarachnoid hemorrhage.

### Neuroimaging studies

All CT studies were scanned by a 64-detector CT system (Brilliance-64, Philips Healthcare) and a power injector was used to administer a bolus of 50 mL contrast at a 4 mL/s flow rate for CTA acquisition. All magnetic resonance (MR) studies were performed by two 1.5 T MR imaging systems (Tim CT and Avanto, Siemens) with 20 slices covering the entire brain (matrix 204-224 × 256-320; field of view 20-23 cm; slice thickness 5 mm, interslice gap 2 mm). Magnetic resonance angiography was obtained using a 3-dimensional time-of-flight MR technique with maximum intensity projection (MIP) (matrix 256 × 256; field of view 18 cm; 110 sections; total coverage 7 cm).

### Treatment and follow up

All patients were suggested to avoid the possible headache triggers. Patients with primary headaches were given analgesics when needed. Patients with RCVS were treated with 30 mg nimodipine four times a day. No patient received intravenous nimodipine. In December 2013, we contacted each patient by telephone to determine whether or not any relapse had occurred. The study protocol was approved by the Institutional Review Board of the hospital.

### Statistics

SPSS 18.0 for Windows (SPSS Inc., Chicago, IL, USA) was used for statistical analyses. Descriptive data were presented as mean ± standard deviation or percentages. Due to the relatively small sample size, non-parametric methods of Fisher’s exact test for categorical data and the Mann Whitney U test for continuous measures were used. All calculated *P*-values were two-sided and significance was defined as a *P*-value < 0.05.

## Results

**Figure 1:**
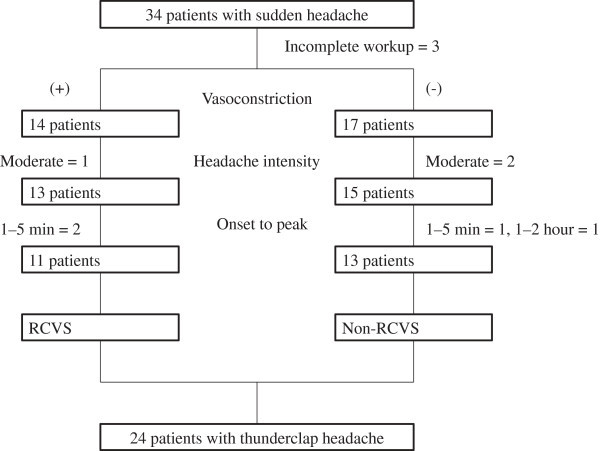
**Screening scheme for patients with sudden headache.** Moderate headache intensity refers to 4-6 on a 0-10 numerical rating scale. RCVS: reversible cerebral vasoconstriction syndrome.

**Figure 2:**
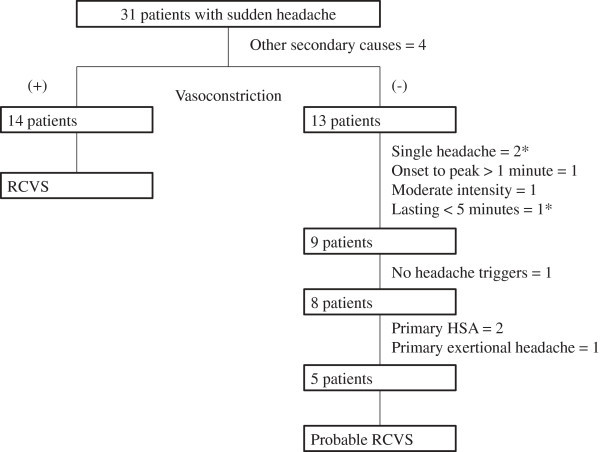
**Test of the ICHD-3 criteria (beta version) in patients with sudden headache.** *One patient had single headache lasting < 5 minutes. Moderate headache intensity refers to 4-6 on a 0-10 numerical rating scale. ICHD: International Classification of Headache Disorders; RCVS: reversible cerebral vasoconstriction syndrome; HSA: headache associated with sexual activity.

From July 2010 to June 2013, 34 consecutive patients presented with SH to our headache clinic. Three patients were excluded for either withdrawal or refusing further diagnostic workup. Twenty-four (77.4%) of the 31 screened patients fulfilled the criteria of TCH (Figure 1). The final diagnoses were listed in Table 2. Of note, 45.2% of SH patients and 45.8% of TCH patients exhibited RCVS. Secondary causes other than RCVS were recorded in 12.9% and 8.3% of patients in the SH and TCH groups, respectively. The ratios of RCVS in patients with “benign TCH”, i.e., excluding patients with other secondary causes, were 51.9% (14 of 27 SH patients) and 50% (11 of 22 TCH patients). Of patients with primary headaches, patients with multiple triggers could not be categorized to any existing diagnoses of ICHD-2. These patients reported either sexual activity or bathing as triggers, and thus could not fulfill the ICHD-2 criteria of primary cough headache (triggered by cough, straining or Valsalva maneuver). We tested the new ICHD-3 criteria (beta version) in our patients (Table 1) (Figure 2). Of the six patients with multiple triggers, five (83.3%) could fulfill the criteria of probable RCVS. Nevertheless, these patients could be categorized as having probable RCVS by the ICHD-3, beta version (Table 1) [14]. The ratios of RCVS and probable RCVS in patients with “benign TCH” would thus be even higher (SH: 70.4%, TCH: 72.7%).

**Table 2:** Final diagnoses of patients with SH and TCH

**Diagnosis**	**Patient number (%)**	
	**SH, n = 31**	**TCH, n = 24**
RCVS	14 (45.2%)	11 (45.8%)
Primary headaches	13 (41.9%)	11 (45.8%)
Multiple triggers	6 (19.4%)	5 (20.8%)
Primary HSA	4 (12.9%)	3 (12.5%)
Primary exertional headache	2 (6.5%)	2 (8.3%)
Primary TCH	1 (3.2%)	1 (4.2%)
Other secondary causes	4 (12.9%)	2 (8.3%)
Chiari malformation	1 (3.2%)	1 (4.2%)
Moyamoya syndrome	2 (6.5%)	1 (4.2%)
Subarachnoid hemorrhage	1 (3.2%)	0

SH: sudden headache; TCH: thunderclap headache; RCVS: reversible cerebral vasoconstriction syndrome; HSA: headache associated with sexual activity.

### Demographics

The demographic and clinical profiles of both SH and TCH patients are listed in Table 3. A trend was observed of patients with RCVS being more likely female (SH: 85.7% vs. 52.9%, *P* = 0.068; TCH: 81.8% vs. 53.8%, *P* = 0.211). Patients with RCVS were older than non-RCVS patients (SH: 50.8 ± 9.3 years vs. 40.8 ± 10.0 years, *P* = 0.006; TCH: 49.4 ± 10.0 years vs. 42.6 ± 10.3 years, *P* = 0.092). In the RCVS group, female patients were significantly older than male patients, but the number of the latter group was very small (women: 53.8 ± 5.8 years, n = 12; men: 33.0 ± 4.2 years, n = 2; *P* = 0.028).

**Table 3:** Demographic and clinical profiles of patients with sudden and thunderclap headaches

	**Sudden headache**				**Thunderclap headache**			
	**Total**	**RCVS**	**Non-RCVS**	**P**	**Total**	**RCVS**	**Non-RCVS**	**P**
	**(n = 31)**	**(n = 14)**	**(n = 17)**		**(n = 24)**	**(n = 11)**	**(n = 13)**	
Age (years)	45.3 ± 10.7	50.8 ± 9.3	40.8 ± 10.0	0.006	45.7 ± 10.5	49.4 ± 10.0	42.6 ± 10.3	0.092
Female	21 (67.7%)	12 (85.7%)	10 (58.8%)	0.132	16 (66.7%)	9 (81.8%)	8 (61.5%)	0.386
Previous headache	17 (54.8%)	10 (71.4%)	7 (41.2%)	0.149	14 (58.3%)	8 (72.7%)	6 (46.2%)	0.240
Precipitating factors				0.304				0.576
None	25 (80.6%)	9 (64.3%)	16 (94.1%)		19 (79.2%)	7 (63.6%)	12 (92.3%)	
Vasoactive substances	4 (12.9%)	3 (21.4%)	1 (5.9%)		3 (12.5%)	2 (18.2%)	1 (7.7%)	
Postpartum	0	0	0		0	0	0	
Headache characters								
Intensity	8.4 ± 1.7	8.6 ± 1.9	8.3 ± 1.6	0.375	8.7 ± 1.2	8.8 ± 1.5	8.5 ± 0.9	0.502
Location				1.0				1.0
Bilateral	17 (70.8%)	7 (70%)	10 (71.4%)		14 (70.0%)	6 (66.7%)	8 (72.7%)	
Duration				0.007				0.004
< 1 hour	14 (51.9%)	3 (23.1%)	11 (78.6%)		10 (45.5%)	1 (10.0%)	9 (75.0%)	
Single attack	4 (12.9%)	0	4 (23.5%)	0.107	2 (15.4%)	0	2 (8.3%)	0.482
Lingering pain	12 (38.7%)	6 (42.9%)	6 (35.3%)	0.724	10 (41.7%)	5 (45.5%)	5 (38.5%)	1.0
Triggers								
Sexual activity	9 (29.0%)	2 (14.3%)	7 (41.2%)	0.132	7 (29.2%)	2 (18.2%)	5 (38.5%)	0.386
Exertion^§^	5 (16.1%)	0	5 (29.4%)	0.048	4 (16.7%)	0	4 (30.8%)	0.098
Valsalva maneuver^§^	14 (45.2%)	8 (57.1%)	6 (35.3%)	0.289	10 (41.7%)	6 (54.5%)	4 (30.8%)	0.408
Emotion^§^	3 (9.7%)	1 (7.1%)	2 (11.8%)	1.0	3 (12.5%)	1 (9.1%)	2 (15.4%)	1.0
Bathing*	6 (19.4%)	6 (42.9%)	0	0.004	4 (16.7%)	4 (36.4%)	0	0.031
Cough^§^	2 (6.5%)	0	2 (11.8%)	1.0	1 (4.2%)	0	1 (7.7%)	1.0
Others	6 (19.4%)	5 (35.7%)	1 (5.9%)	0.067	4 (16.7%)	3 (27.3%)	1 (7.7%)	0.300
No triggers	3 (9.7%)	1 (7.1%)	2 (11.8%)	1.0	2 (8.3%)	0	2 (15.4%)	0.482
Possible neurological symptoms	3 (9.7%)	2 (14.3%)	1 (5.9%)	0.576	1 (4.2%)	1 (9.1%)	0	0.458
Abnormal CT or MRI^†^	5 (16.1%)	3 (21.4%)	2 (11.8%)	0.636	4 (16.7%)	3 (27.3%)	1 (7.7%)	0.300
Lumbar puncture	3 (9.7%)	3 (21.4%)	0	0.081	2 (8.3%)	2 (18.2%)	0	0.199
Angiography	8 (25.8%)	5 (35.7%)	3 (17.6%)	0.412	7 (29.2%)	5 (45.5%)	2 (15.4%)	0.182

Values presented are either mean ± standard deviation or number (%).

RCVS: reversible cerebral vasoconstriction syndrome; CT: computed tomography; MRI: magnetic resonance imaging.

^§^Valsalva maneuver includes defecation, urination, sneezing, bending, heavy lifting and related exercise. Laughing and crying are categorized as “emotion.” Exertion includes exercises not specifically involving Valsalva maneuvers. Cough includes only coughing but not straining or Valsalva maneuvers.

*Bathing includes also showering or exposure to water.

^†^Abnormal CT or MRI refers to changes other than vasospasm or moyamoya vessels.

### Precipitating factors

No patient in our study was in the postpartum state. The patients’ medication history was carefully obtained but we did not observe any patient use of recreational drugs, selective serotonin reuptake inhibitors, triptans, immunosuppressants or even recent use of herbal medication. All patients using possible vasoactive substances had been using cough medication. For some patients, to determine which drugs they had used was simply not feasible. We thus adopted a loose guideline that use of any cough medication just before occurrence of headaches would be recorded. Since use of cough medication was very common, and not all cough medications precipitate RCVS, the percentage was likely over-estimated.

### Headaches

As for the headache characteristics, patients with RCVS were less likely to exhibit short headache duration of < 1 hour (SH: 23.1% vs. 78.6%, *P* = 0.007; TCH: 10% vs. 75%, *P* = 0.004). The mean headache duration was not presented because most patients described their headache duration as a range e.g. several minutes (the shortest) or up to one day (the longest). We tried different cutoff points including half an hour and four hours but the best one to differentiate the two groups was one hour. Also, patients with RCVS were more likely to report bathing as their headache trigger (SH: 42.9% vs. 0%, *P* = 0.004; TCH: 36.4% vs. 0%, *P* = 0.031). On the other hand, patients with non-RCVS were more likely to report exertion as a trigger, but significance of this difference existed only in the SH sample (SH: 0% vs. 30.8%, *P* = 0.098; TCH: 0% vs. 29.4%, *P* = 0.048). Three patients complained of possible neurological symptoms during headache attacks (Table 3). This included bilateral leg numbness (RCVS), restlessness sensation of legs (RCVS with cortical SAH), and left palm numbness (moyamoya syndrome). All symptoms subsided before the patients called upon our clinic and no patients demonstrated abnormal neurological examination findings.

### Diagnostic workup

**Figure 3:**
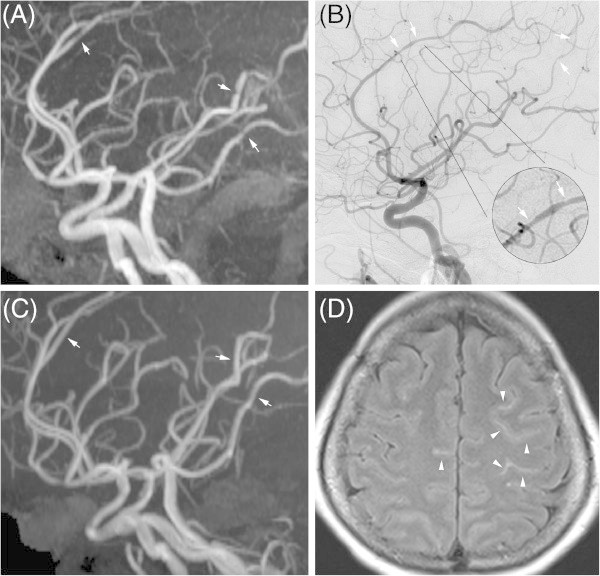
**Imaging findings of reversible cerebral vasoconstriction syndrome.** Multifocal vasoconstriction demonstrated by magnetic resonance angiography (MRA) **(A)** and catheter angiography **(B)**, involving the anterior, middle, and posterior cerebral arteries. Follow-up MRA **(C)** revealed significant interval resolution of the previous lesions. Axial fluid attenuated inversion recovery (FLAIR) imaging revealed linear hyperintensity lesions in the sulci of the bilateral frontal lobes **(D)**.

Three patients with RCVS exhibited multifocal linear high-intensity signals along the cortical sulci and subarachnoid space (Figure 3). The neuroradiologist (Kuo KH) was more in favor of cortical SAH though the possibility of hyperintense vessels may not be excluded [15]. All of these patients received CT prior to their MRI exams and only one patient exhibited comparable hyperdense lesions. Catheter angiography was performed in each patient and no aneurysm was found. Follow-up MRI revealed resolution of the signals. One patient received lumbar puncture at emergency department two weeks before she presented to our clinic. The open and close pressures were 230/195 mmH_2_O with the presence of red blood cells (143 cells/mm^3^). The data were regarded as due to a traumatic tap and no further workup was performed. In our study, total three RCVS patients had received lumbar puncture while normal results were found in the other two patients.

### Treatment and follow up

All patients recovered without neurological sequelae, including the patient with aneurysmal SAH received surgery. We also did not encounter any patients who develop new neurological symptoms or signs after their visits. The response to nimodipine in patients with RCVS was not presented because the adherence to headache diary was low. We contacted the patients by phone in December 2013; at this time, no patient had relapse of similar severe headaches or been diagnosed with SAH or sudden death. One patient with RCVS and one patient with primary exertional headache reported incomplete recovery and mild residual headache. Within the inclusion period, one patient reported relapse 22 months after his first attack. This patient was diagnosed with primary HSA after the first attack; however, vasoconstriction was noted during the relapse and the diagnosis was subsequently changed to RCVS.

## Discussion

In our study, 14 (45.2%) of the 31 patients with SH and 11 (45.8%) of the 24 patients with TCH had RCVS. Over past decades, several studies have recruited patients with SH, focusing on the identification of SAH and sentinel headache (Table 4). A significant proportion of patients exhibited SAH and other secondary causes, but more than one-half of the patients in these studies did not receive a definite diagnosis. It has been postulated that RCVS accounts for most of these “benign TCH” cases [13]. Our results show that about one-half of these patients exhibit RCVS. Due to a lack of similar studies, to the best of our knowledge, we compared our results with studies addressing related headache syndromes. In a study of 30 patients with HSA, 18 (60%) patients exhibited RCVS, 10 (33.3%) exhibited primary HSA and 2 (6.7%) exhibited other secondary causes (SAH and basilar artery dissection) [16]. In another study of 21 patients with bath-related TCH, 13 (62%) patients exhibited RCVS and no other secondary causes were identified [17]. The target populations in the above studies varied from that of our study: patients in the above studies were recruited mainly based on specific triggers (sexual activity and bathing, respectively), while our patients presented with SH or TCH, with or without triggers. In conclusion, our study provides direct evidence that RCVS is a common and likely under-recognized cause of SH and TCH.

**Table 4:** Summary of studies on patients with sudden headache

	**Current study**	**Perry**[5]	**Landtblom**[4]	**Linn**[3]	**Harling**[26]
SAH	3.3%	6.2%	11.3%	25%	71.4%
No diagnosis	19.4%	57.7%	73.0%	62.8%	2.0%
	(3.3%)*				
Onset	sudden	< 1 hour	sudden	< 1 minute	sudden
Intensity	NS	NS	NS	Severe	NS
Duration	> 1 minute	NS	NS	> 1 hour	NS
Onset to visit	< 30 days	< 14 days	NS	NS	NS
Settings	Clinic	Emergency department	Emergency department	Clinic and emergency	In-hospital
Follow up	6-42 months	6 months	12 months	12 months	18-30 months
Further SAH	0	0	0	0	0

SAH: subarachnoid hemorrhage; NS: not specified.

*When we conducted the study, patients with multiple triggers could not be classified by the criteria of International Classification of Headache Disorders, 2nd edition (ICHD-2) (Table 1). However, five of these six patients may be categorized as probable reversible cerebral vasoconstriction syndrome by the criteria of ICHD-3, beta version (Table 1) (Figure 2).

Demographic data in our RCVS patients recaptured an obvious pattern reported in other studies with larger sample sizes: a majority of older female and a minority of younger male patients. In our study, around 80% of patients with RCVS were female, compared with 64.2% to 89.6% in previous studies [9,11,18]. Female RCVS patients were typically aged in their late forties or fifties, while male patients were aged in their thirties (women: 53.8 ± 5.8 years, men: 33.0 ± 4.2 years). The mean age of our female RCVS patients seemed slightly older than those in other studies (women: 44.2-49.7 years, men: 34.7-34.9 years) [9-11]. We propose that this difference is due to the absence of a third group of RCVS patients in our study: younger women in the postpartum state. This proposition is likely supported by the larger standard deviation in studies with higher ratios of postpartum female [9,11].

The clinical profiles of our RCVS patients differed from previous studies in several aspects. First, our patients demonstrated more favorable outcomes. Only a few patients presented with subtle neurological symptoms, and no patients exhibited focal neurological signs or seizure throughout the course of the study. We did not observe any complications of cerebral infarction, intracerebral hemorrhage, or posterior reversible encephalopathy syndrome. Second, most of our RCVS patients were spontaneous (78.6%), i.e., without precipitants. There was no patient in the postpartum state. All patients with possible vasoactive substances used cough medicine and the percentage was probably over-estimated (please refer to Precipitating factors paragraph of Results). We encountered only one Vietnamese patient who reported use of ecstasy one month after childbirth but she was seen before the initiation of the study and thus not included [19]. This was in contrast with the use of recreational drugs (especially cannabis) in French study or serotonergic drugs (selective serotonin reuptake inhibitors and triptans) in American study [9,11]. In general, the above clinical profiles were similar to another Taiwanese study but differed from French and American cohorts [9-11]. The discrepancy may be attributed to the clinic-based settings of both Taiwanese studies, in comparison with emergency or inpatient settings of other studies, i.e. patients with complications were more likely to call upon emergency service and to be admitted. It is also possible that the benign course of our RCVS patients were related to the absence of postpartum state and cannabis use, as both have been associated with stroke and poorer outcome [20,21]. However, another study from the same French group reported women and migraine history, instead of postpartum or vasoactive substances, were associated with intracranial hemorrhage in RCVS patients [22]. Two recent retrospective studies of RCVS do not identify any precipitants as predictors of outcome. Of note, the target in one study was clinical worsening (rather than final outcome), while the other study was small in sample size (n = 10) [23,24].

In our study, patients with RCVS were less likely to have short headache duration < 1 hour (Table 3). The duration of RCVS ranges from 5 minutes to 36 hours with a mean of 5 hours in one study, and a median of 3 hours in another study [9,10]. The duration of primary headaches vary in a wide span, by the definition of ICHD-2 [6]. For primary TCH, the duration range is 1 hour to 10 days and for primary exertional headache, the duration range is 5 minutes to 48 hours. Nevertheless, a study of 596 adolescent patients with primary exertional headache reported 467 (78.4%) patients had headache duration < 1 hour [25]. The duration of primary cough headache (1 second to 30 minutes) is significantly shorter than the duration of RCVS; however, only two (11.8%) of the non-RCVS patients in our study reported cough as the trigger (Table 3). Another study of HSA showed no difference in headache characteristics, including duration, between patients with primary HSA and RCVS [16]. In general, fewer patients with RCVS had short headache duration (< 1 hour) compared to non-RCVS patients; however, the headache duration may vary significantly in the latter group.

Among the various triggers, bathing (including showering and water exposure) was more frequently associated with RCVS in both SH and TCH cohorts. It has been reported that RCVS was noted in 13 (62%) of 21 patients with bath-related TCH [17]. In our study, all patients reported bathing as a trigger were in the RCVS group (100%, n = 6). Bathing is unique among RCVS triggers, in that it is not associated with the Valsalva maneuver or emotion. In contrast to bathing, exertion was associated with non-RCVS diagnoses, but the significance of this association was borderline, and only in the SH sample (SH: *P* = 0.048; TCH: *P* = 0.098). Further studies are necessary to validate the associations between different triggers and RCVS.

Although TCH was first used to describe the headache associated with an unruptured aneurysm [1], only one (3.1%) patient with SAH was noted in our study. The studies focused on SAH and sentinel headache on patients with SH showed that 6.2-25% of them exhibited SAH, except for the earliest study, which identified 71.4% of patients exhibiting SAH (Table 4) [3-5,26]. Compared with a very modest decline of SAH incidence over recent decades, the dramatic decrease of SAH ratios in these studies was confusing [27]. We proposed that the low ratio of SAH in our patients may be due to the following reasons. First, the definition of SH, unlike that of TCH, was not well-determined and varied with each study (Table 4). Second, previous studies either recruited patients from an emergency department only or from both emergency and outpatient clinics, while our study was strictly clinic-based. Given the low rates of patients receiving lumbar puncture and catheter angiography in our study, the possibility of missed SAH may not be excluded. Nevertheless, based on the high sensitivity of the modern CT to detect SAH and CTA/MRA to detect aneurysm and the fact that no patients developed SAH during at least 6 months of follow-up, the contribution of missed SAH to the low SAH percentage in our study may be minor [17,28,29].

This study has limitations. First, the sample size is relatively small and further study with a larger population is needed. Second, as stated above, the definition of SH and the clinical setting were not consistent across studies and the ratios of RCVS in these patients may change accordingly. In this study, we included patients with a new SH of possible vascular origins while excluded those with typical clinical presentations suggesting other primary or secondary headaches. This may carry a potential risk of missing SAH or RCVS patients presenting with typical features of other headaches. We also excluded patients with recurrent SH or TCH. Interpretation and generalization of the data should be handled with caution. Third, the percentage of patients receiving catheter angiography was low. Although CTA and MRA have been widely accepted for detection of vasoconstriction in patients with RCVS, catheter angiography is still the gold standard [7,18]. Besides, vasoconstriction has been reported to elude primary detection, and serial repetition may be necessary [9,18]. Therefore, the percentage of RCVS may be underestimated. This observation did not change—and may perhaps strengthen—our conclusion that RCVS is a common cause of SH and TCH. The low angiography rate may also contribute to the result that arterial dissection in patients with RCVS was not observed [30]. Fourth, the percentage of RCVS patients receiving lumbar puncture was also low (SH: 21.4%, TCH: 18.2%). As stated above, we could not exclude the possibility of missed SAH, but the chance may be low. Fifth, several rare causes of TCH were not excluded properly. Without MR venography, cerebral venous sinus thrombosis may be ignored, but the neuroradiologist denied any related findings by CT or MRI in all patients. Without routine lumbar puncture, meningitis may be undetected; however, none of the patients exhibited fever or neck stiffness. We did not screen pheochromocytoma, though no patients exhibited uncontrolled hypertension. Altogether, these diseases were rare in patients with isolated TCH, and we were convinced that the ratio of RCVS may not change significantly [13].

## Conclusions

In this study, we provided direct evidence of RCVS as a common cause of SH and TCH. Demographic data in our RCVS patients recaptured a typical pattern reported in other studies: a majority of older female and a minority of younger male patients. The clinical profiles were similar to another clinic-base study from Taiwan but differed from other studies with emergency or inpatient settings. Compared to non-RCVS patients, patients with RCVS were older and had longer headache duration. They were more likely to cite bathing but less likely to cite exertion as triggers. Reversible cerebral vasoconstriction syndrome is a clinical emergency linked with potential clinical worsening, morbidity and even mortality [23,24]. Systemic examination of cerebral vessels should be adopted in these patients, so that they may benefit from potential treatments such as avoidance of triggers and use of nimodipine.

## Abbreviations

CT: Computed tomography; CTA: Computed tomography angiography; HSA: Headache associated with sexual activity; ICHD: International Classification of Headache Disorders; MR: Magnetic resonance; MRA: Magnetic resonance angiography; MRI: Magnetic resonance imaging; RCVS: Reversible cerebral vasoconstriction syndrome; SAH: Subarachnoid hemorrhage; SH: Sudden headache; TCH: Thunderclap headache.

## Competing interests

The authors declare that they have no competing interests.

## Authors’ contributions

CYC collected the data, analyzed the data and participated in manuscript preparation. KKH was responsible for the imaging protocols, interpretation and preparation of Figure 3. LTH took charge of the whole study, especially the ideation, recruitment of patients and manuscript preparation. All authors read and approved the final manuscript.
